# The Therapeutic Potential of *Harpagophytum procumbens* and *Turnera subulata* and Advances in Nutraceutical Delivery Systems in Neurodegenerative Diseases

**DOI:** 10.3390/ph17050660

**Published:** 2024-05-20

**Authors:** Antonio Carlos Vital Júnior, Mikaelly Batista da Silva, Shênia Santos Monteiro, Matheus Augusto de Bittencourt Pasquali

**Affiliations:** 1 Post-Graduate Program in Biochemistry and Molecular Biology, Federal University of Rio Grande do Norte, Natal 59078-970, Brazil; 2 Center for Technology and Natural Resources, Federal University of Campina Grande, Campina Grande 58429-900, Brazil; 3 Graduate Program in Food Engineering, Federal University of Campina Grande, Campina Grande 58429-900, Brazil

**Keywords:** bioactive compounds, blood–brain barrier, nanotechnology, nutraceuticals

## Abstract

This review article covers the therapeutic potential of the plants *Harpagophytum procumbens* and *Turnera subulata* in the treatment of neurodegenerative diseases. Despite the recognition of their beneficial properties, there is notable shortage of specific clinical and in vitro studies on these species regarding neurodegenerative diseases. Compounds such as harpagosides and vite-xin-2-O-rhamnoside, found in *Harpagophytum procumbens* and *Turnera subulata*, respectively, as well as other antioxidants and anti-inflammatory agents, are associated with mechanisms of action that involve reducing oxidative stress and modulating the inflammatory response, indicating their therapeutic potential in these pathologies. Additionally, the use of nutraceuticals derived from medicinal plants has emerged as a promising approach, offering natural therapeutic alternatives. However, the pressing need for studies focusing on the pharmacokinetics, safety, and pharmacological interactions of these extracts for the treatment of neurodegenerative diseases is emphasized. This review also evaluated advances in nutraceutical delivery systems, highlighting technological innovations that can optimize the precise delivery of these compounds to patients. Such findings highlight the gaps in the study of these plants for the treatment of neurodegenerative diseases and, at the same time, the potential for opening new perspectives in the treatment of neurodegenerative diseases, providing expectations for innovative solutions in this critical domain of medicine.

## 1. Introduction

Neurodegenerative diseases are characterized by the selective and symmetric loss of neurons in the motor, sensory, or cognitive systems. The most common neurodegenerative diseases, such as Alzheimer’s disease (AD), Parkinson’s disease (PD), multiple sclerosis (MS), and Huntington’s disease (HD), stand out for their clinical manifestations, including extrapyramidal and pyramidal movement disorders, as well as cognitive and behavioral disturbances [1]. It is worth noting that multiple sclerosis (MS) is not only an autoimmune disease but also a neurodegenerative disease triggered by an inflammatory attack of the central nervous system (CNS). Neurodegeneration, which includes neuronal cell death, apoptosis, necrosis, and virtual hypoxia, develops alongside inflammation and demyelination [2,3,4].

The overall mortality and morbidity of these diseases are increasing due to the aging of the population. However, conditions such as multiple sclerosis have a prevalent incidence in individuals between 20 and 40 years old [5]. Although evidence suggests that DNA damage, oxidative stress, mitochondrial dysfunction, neuroinflammation, and other risk factors play a role in neurodegenerative diseases, the underlying molecular mechanisms remain poorly understood, and there is currently no cure for most of these conditions, only symptomatic drug administration [6,7].

Currently, developed and approved medications are used to treat the symptoms of neurodegenerative diseases but cannot reverse the clinical condition of patients. Due to this limitation, there has been a growing interest within the scientific community in investigating the real potential and mechanisms of action of plants used in natural medicine for treating these diseases, especially AD and PD. *Harpagophytum procumbens* and *Turnera subulata* stand out among the traditionally used medicinal plants that demonstrate potent biological actions. With scientific reports indicating the benefits of the components present in *Turnera subulata*, such as its potential antioxidant, anti-inflammatory, and anti-angiogenic properties, it is believed that the plant may offer beneficial properties. Although there is still no direct scientific evidence, and further studies need to be conducted to prove its efficacy [8], these plants have attracted the attention of researchers because they present promising molecules derived from their secondary metabolisms, such as flavonoids, saponins, and alkaloids, which may contribute to the treatment of neurodegenerative diseases in the future, which justifies the need to develop work along this line.

In this context, it is important to highlight that the use of plant bioactive extracts is considered an excellent prospect for obtaining new neuroprotective medications for the treatment of PD and AD. Additionally, it also presents potential for the development of other neuroprotective, therapeutic, or preventive strategies, such as their use in nutraceutical and phytotherapeutic products.

While plant extracts are advantageous preparations, they are unstable and susceptible to chemical or microbiological degradation. Long-term storage, in particular, poses some challenges as phytochemicals are negatively affected by physicochemical factors such as exposure to light, heat, oxygen, and humidity, which lead to alterations in their chemical composition and adversely impact their biological activities [9]. Furthermore, the effectiveness of numerous therapeutic agents intended for the clinical management of neurodegenerative diseases via oral and parenteral routes is often compromised by insufficient drug transport to the brain. This limitation primarily arises from the obstacles presented by the blood–brain barrier (BBB), the blood–cerebrospinal fluid barrier, and the brain–cerebrospinal fluid barrier. These barriers constitute formidable obstacles to drug delivery to the brain and the successful treatment of neurodegenerative diseases [10].

To overcome these barriers, advances in nanotechnology have led to the development of highly effective drug delivery systems that can be customized to transport biomolecules through the BBB and deliver them to the brain. Furthermore, nanoparticles can protect the drug or loaded bioactive compound against enzymatic degradation and immunological clearance, resulting in an extended half-life and improved bioavailability [10].

Overall, the search for effective and innovative treatments for neurodegenerative diseases has been a top priority in medical research. In this context, the investigation of the therapeutic potential of medicinal plant extracts, from plants such as *Harpagophytum procumbens* and *Turnera subulata*, has been gaining prominence. These plants, with a long history of use in traditional medicine, have piqued the interest of the scientific community due to their beneficial properties. Furthermore, advances in nanotechnology and innovative delivery systems for bioactive compounds offer a promising approach for the effective and targeted administration of these extracts to the brain, with the potential to revolutionize the treatment of neurodegenerative diseases. In this review article, we explore the therapeutic benefits of these plants and try to highlight studies that address the effects of innovative delivery systems with a focus on neurodegenerative diseases. However, studies are scarce and insufficient for portraying the mechanisms of action and delivery systems of nutraceuticals aimed at treating these diseases. Therefore, despite this fact, our study can contribute to opening up new horizons and encourage researchers to seek new forms of treatment for these debilitating conditions.

## 2. Therapeutic Properties of *Harpagophytum procumbens* and *Turnera subulata*

### 2.1. Harpagophytum procumbens

**Figure 1:**
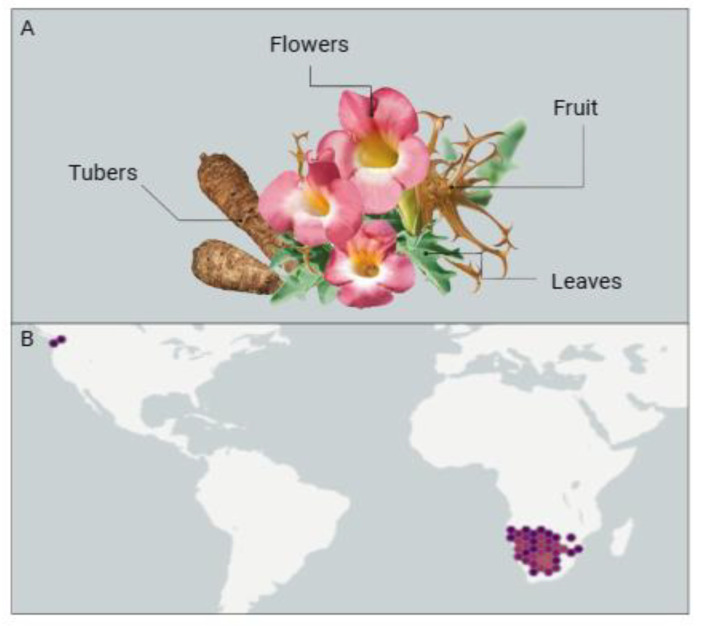
(**A**) Morphological representation, encompassing flowers, leaves, fruits, and tubers, and (**B**) geographical occurrence of *Harpagophytum procumbens* [12].

*Harpagophytum procumbens*, belonging to the Pedaliaceae family, is a perennial tuberous plant considered invasive, known for its visually striking fruits, as can be seen in Figure 1A. These fruits have numerous long, pointed projections resembling grappling hooks and feature two straight spines on the upper surface, which earned it the common name “Devil’s Claw” for the Harpagophytum genus [11].

The plant’s flowers are tubular and display a deep mauve color with a throat that is yellow and white, while its leaves are bluish-green and typically irregularly divided into multiple lobes (Figure 1A) [11].

Its annual creeping stems can reach up to 2 m in length and emerge after the first rains but wither during the dry season or periods of drought. These stems originate from a primary tuber, whose main root can reach up to 2 m deep. The plant also produces several secondary tubers branching out from the primary tuber [13].

*Harpagophytum procumbens* is found in some South African countries (Figure 1B). This plant is commonly found in regions with low annual precipitation, typically between 150 and 500 mm per year, in the characteristic red sandy soils of the Kalahari Desert, being more prevalent in open areas subject to overgrazing since it cannot effectively compete with grasses [11,13]. To survive during extended periods of severe drought, the plant stores water in secondary tubers [13]. These secondary tubers are harvested for their medicinal properties.

#### 2.1.1. Anti-Inflammatory Activity

For generations, indigenous cultures have harnessed the therapeutic properties of the *Harpagophytum procumbens* plant, particularly in the treatment of inflammatory and arthritic diseases. This ancient use has sparked increasing interest within the scientific community, leading to research on its tuberous roots, extracts, and oils [14].

*Harpagophytum procumbens* is known for the presence of various iridoid glycosides, acetylated phenolic glycosides, and terpenoids [15]. Notable among them are harpagoside and harpagide, which have been the most extensively studied iridoid glycosides in research associating their properties with anti-inflammatory, analgesic, antioxidant, and antiarthritic actions [16]. Furthermore, phenolic glycosides can also be found, such as 8-feruloylharpagide, verbascoside, leucosceptoside, and pagoside [17,18,19].

These phytochemicals suppress the expression of cyclooxygenase-2 (COX-2) and prevent the synthesis of proinflammatory cytokines such as tumor necrosis factor-alpha (TNF-α) and interleukin-1 beta (IL-1β) [20,21]. The suppression of these inflammatory mediators leads to reductions in the inflammatory response and the pain associated with various diseases, including osteoarthritis and rheumatoid arthritis [22].

In a study conducted by Fiebich et al. [23], it was found that a *Harpagophytum procumbens* extract significantly suppressed TNF-α and interleukin 6 (IL-6) levels at concentrations of 100 μg/mL and 200 μg/mL, respectively. Furthermore, according to Brendler [24], an extract exhibited significant anti-inflammatory effects, reducing TNF-α levels at EC50 concentrations of 116 ± 8.2 µg/mL and 49 ± 3.5 µg/mL.

In addition to its anti-inflammatory properties, *Harpagophytum procumbens* demonstrates analgesic effectiveness, alleviating the pain associated with inflammatory and degenerative diseases. This analgesic effect is related to the production of COX-2 and prostaglandin E2 (PGE2), along with the inhibition of prostaglandin synthesis [17,25,26]. Therefore, for individuals suffering from conditions such as osteoarthritis, harpagosides, particularly harpagoside, and harpagide, play a crucial role in modifying pain perception and, thus, improving the clinical condition of patients [27,28].

As mentioned earlier, the analgesic and anti-inflammatory properties of *Harpagophytum procumbens*, when combined, reduce pain, stiffness, and inflammation in affected joints, playing a fundamental role in its antiarthritic activity [29]. Furthermore, the plant’s chondroprotective properties assist in preserving cartilage integrity and may slow disease progression [30].

#### 2.1.2. Antioxidant Activity

Antioxidant activity plays a crucial role in reducing oxidative stress and the resulting tissue damage [31]. The presence of polyphenolic chemicals, such as flavonoids (like kaempferol and luteolin) and phenolic acids (e.g., cinnamic, caffeic, and chlorogenic acids), is largely responsible for the plant’s antioxidant properties [32,33,34].

These compounds contribute to the plant’s antiaging and cytoprotective properties, helping eliminate free radicals and reduce oxidative damage. Additionally, the antioxidants found in *Harpagophytum procumbens* can support cellular homeostasis and reduce the risk of the chronic diseases related to oxidative stress [35].

#### 2.1.3. Neuroprotective Activity

Given that chronic neuroinflammation is one of the biological factors contributing to neurodegeneration and cognitive aging, plants like *Harpagophytum procumbens*, with their anti-inflammatory and antioxidant properties, have been extensively researched for their potential use in the treatment of these diseases [22]. These compounds, primarily found in the plant’s secondary tubers, help combat the oxidative stress caused by various endogenous and exogenous sources and consequently alleviate the symptoms of diseases characterized by the exacerbated production of reactive oxygen/nitrogen species in their etiology, such as PD and AD [36,37,38,39].

In Table 1, we provide a concise overview of the remarkable therapeutic effects of *Harpagophytum procumbens* regarding its neuroprotective action.

Although *Harpagophytum procumbens* has been traditionally used to treat various conditions such as pain, inflammation, and digestive problems [19,44], the current scientific literature has focused on preclinical studies to validate its efficacy and safety in medicine. The clinical translation of using this plant in the treatment of neurodegenerative diseases faces considerable challenges. The identification of biomarkers that can indicate treatment efficacy is crucial, especially considering the neuroinflammation associated with the pathogenesis of these diseases.

Neuroinflammation, triggered by the activation of glial cells in response to pathogenic agents, is associated with neurodegenerative diseases. This response results in the release of various neuroinflammatory markers, including TNF-α, IL-1β, IL-10, nitric oxide (NO), and COX-2, among others [45]. Cyclooxygenases (COXs) are enzymes linked to these diseases, especially COX-2, which is expressed in certain cell populations of the brain. Studies indicate that its expression varies at different stages of neurodegenerative diseases, such as AD [46,47].

In vitro, researchers investigated the potential of compounds from *Harpagophytum procumbens* to inhibit COX-2 expression. These studies have highlighted significant anti-inflammatory effects, especially with harpagoside and 8-coumaroylharpagide [48]. The harpagoside fraction was also identified as responsible for inhibiting COX-1 and COX-2 [49]. Additionally, evidence suggests that the plant may have neuroprotective effects, reducing the oxidative stress and gliosis induced by neurotoxic agents [40].

Despite the documented antioxidant and anti-inflammatory properties, the pharmacokinetic information about *Harpagophytum procumbens* is limited, mainly due to the scarcity of in vivo studies. In an in vitro study conducted by Chrubasik et al. [50], it was shown that harpagoside, a key compound in the plant’s attributed biological action, can be hydrolyzed to harpagogenin under acidic conditions that mimic the physiological conditions of the stomach. However, harpagogenin has not been isolated in vivo, likely due to its high reactivity and protein-binding rate. In that study, it was reported that harpagoside has a high octanol-water distribution coefficient of approximately four, which is not dependent on temperature or pH. In artificial gastric fluid, the harpagoside content decreases by about 10% within 3 h, while it remains stable in artificial intestinal fluid for 6 h [50].

The pharmacological interactions of *Harpagophytum procumbens* have not been fully elucidated, although safety studies have not reported significant adverse events in patients treated with the plant. A systematic review conducted by Vlachojannis et al. [51] evaluated 28 clinical trials with *Harpagophytum procumbens*. It was found that none of the double-blind studies reported a higher incidence of adverse events during treatment with *Harpagophytum procumbens* compared to those with treatment with a placebo. Minor adverse events occurred in about 3% of patients, mainly gastrointestinal adverse events. There were no reports of chronic toxicity. Chantre et al. [52] conducted a double-blind, randomized, multicenter study comparing the efficacy and tolerability of a herbal product based on *Harpagophytum procumbens* (435 mg) with diacerein (100 mg/day) in the treatment of patients with knee and hip osteoarthritis over 4 months. The results showed that the frequency of adverse events was significantly lower in the group receiving the *Harpagophytum procumbens* herbal product than in the group receiving diacerein. Therefore, the results of this study showed that the product derived from *Harpagophytum procumbens* is comparable in efficacy and superior in safety to diacerein.

Despite the neuroprotective potential of *Harpagophytum procumbens*, the lack of robust clinical studies and accurate information on dosage and pharmacological interactions necessitates further investigation to fully understand its safety and efficacy.

### 2.2. Turnera subulata

**Figure 2:**
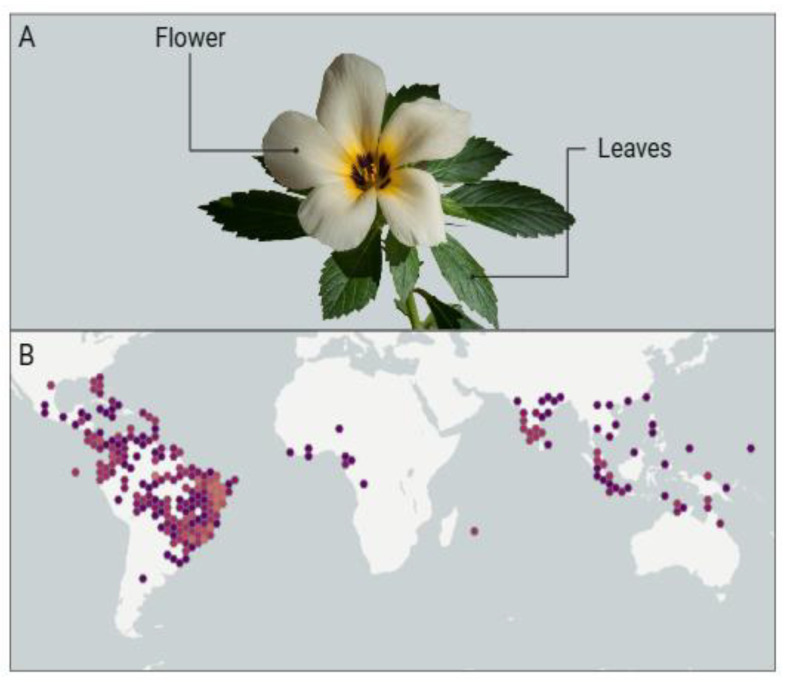
(**A**) Morphological representation, encompassing flowers and leaves, and (**B**) geographic occurrence of *Turnera subulata* [12].

*Turnera subulata*, a plant widely used in traditional medicine for centuries, is a subshrub native to South and Central America, which has also become established in the Indian subcontinent, as illustrated in Figure 2B. This plant is a perennial herb that often develops with strong and dense roots, featuring a cylindrical woody stem that reaches a height of 30 to 80 cm [53].

Its stem is sparingly branched, tomentose, with simple leaves, petiolate, membranous, slightly discolored, elliptical in shape, and serrated margins. *Turnera subulata* flowers are solitary and grow epiphyllous, with the peduncle adnate to the petiole. The calyx, located at the basal portion, is gamosepalous, yellowish-green in color, adorned with subulate bracteoles. Its corolla is dialipetalous, with cream-colored petals at the base and a dark brownish hue [54]. The morphological characteristics of this plant can be observed in Figure 2A.

In the northeastern region of Brazil, *Turnera subulata* plays a crucial role in treating conditions such as amenorrhea and dysmenorrhea. It is also used for the treatment of tumors, flu, and cuts, as well as gastrointestinal and respiratory diseases [53]. This plant is notable for its abundance of antioxidant properties, attributed to the presence of essential oils. Furthermore, its composition includes substances such as steroids, flavonoids, and pheophytin [55].

Despite popular knowledge of its medicinal properties for centuries, the use of *Turnera subulata* as a herbal remedy has been little explored, and its pharmacological properties and biological activity lack further clarification. According to recent studies, the plant’s main biological activities include antianxiety, antioxidant, antibacterial, and wound-healing effects [53,56].

The biological effects related to the antianxiety properties of *Turnera subulata* are widely recognized, as documented by Rebouças et al. [57]. The main contributors to these effects include the flavonoids, terpenoids, and alkaloids from the plant [58]. The ability of *Turnera subulata* to regulate the central nervous system neurotransmitter, gamma-aminobutyric acid (GABA), is the primary cause of its antianxiety effects [59,60]. Additionally, the antioxidants found in the plant play an effective role in eliminating free radicals and reducing oxidative stress, protecting against cellular damage [61,62]. Therefore, due to the antioxidant quality of this plant, the use of plant extracts significantly contributes to the prevention of chronic diseases related to oxidative stress and overall health promotion, as pointed out by Annadurei et al. [63].

Another relevant biological aspect of the compounds isolated from *Turnera subulata* is their ability to inhibit and/or eliminate Gram-positive and Gram-negative bacteria and fungi [64,65,66]. This antimicrobial activity of the plant is related to the presence of polyphenols, terpenoids, and essential oils [67]. The main phenolic compounds related to the antimicrobial activity are vitexin-2-O-rhamnoside, 7-O-beta-glucopyranosyl-4′-hydroxy-5-methoxyisoflavone, and ferulic acid [68,69,70].

Among the previously mentioned pharmacological properties of *Turnera subulata*, its anti-inflammatory property stands out, showing potential for use in the treatment of neurodegenerative diseases. A study conducted by Luz et al. [62] investigated the immunomodulatory effect of the floral and leaf extracts of this plant in an in vitro model of acute inflammation using macrophages. The results obtained revealed that the bioactive molecules of *Turnera subulata* have a promising advantage regarding side effects. In addition, it is essential to note that the extracts evaluated through cell viability assays using murine macrophages (RAW 264.7) exposed to different concentrations (5, 50, 100, and 500 µg/mL) did not cause cytotoxicity, thus minimizing the possibility of unwanted side effects, such as systemic, gastrointestinal, or other health problems related to treatment with the plant. This reinforces the potential safety of using *Turnera subulata* as a therapeutic option for neurodegenerative diseases [62].

In Table 2, the main therapeutic properties related to the herbal use of *Turnera subulata* with potential action in the treatment of neurodegenerative diseases are summarized.

The *Turnera subulata* plant presents therapeutic potential, yet the lack of dedicated research on its pharmacokinetics, safety, and drug interactions represents a significant gap in current studies. Although traditionally used in folk medicine for various purposes, such as treating gastrointestinal problems, anxiety, and insomnia, the scarcity of clinical studies and comprehensive research limits our understanding of its safety and efficacy. Evaluating these aspects is crucial in guiding the appropriate use of *Turnera subulata* in clinical practice and identifying potential interactions with other prescribed medications or supplements. Therefore, further detailed research is needed to fill this knowledge gap and provide solid scientific evidence to support its therapeutic use.

Studies focused on the search for promising sources of treatments through natural resources, such as medicinal plants, constitute one of the essential pillars for the progress and advancement of the nutraceutical field. Given the vast diversity of plant species available, there is an extensive field of exploration of and research on bioactive compounds that can be applied in the treatment and prevention of diseases.

Despite the noticeable increase in research aimed at understanding the role of bioactive compounds from plants, it is increasingly important to reinforce the importance and benefits of these metabolites for our health and quality of life.

## 3. Nutraceutical Delivery Systems

As the life expectancy of the global population continues to increase, there is growing concern about diseases typically associated with aging, including PD, AD, amyotrophic lateral sclerosis (ALS), HD, and MS [72]. These patients experience modifications in the nervous system, including changes in the structural, biochemical, and electrical functions of the brain, spinal cord, and nerves, resulting in a wide range of symptoms such as paralysis, muscle weakness, lack of coordination, seizures, confusion, pain, and changes in consciousness [73].

**Figure 3:**
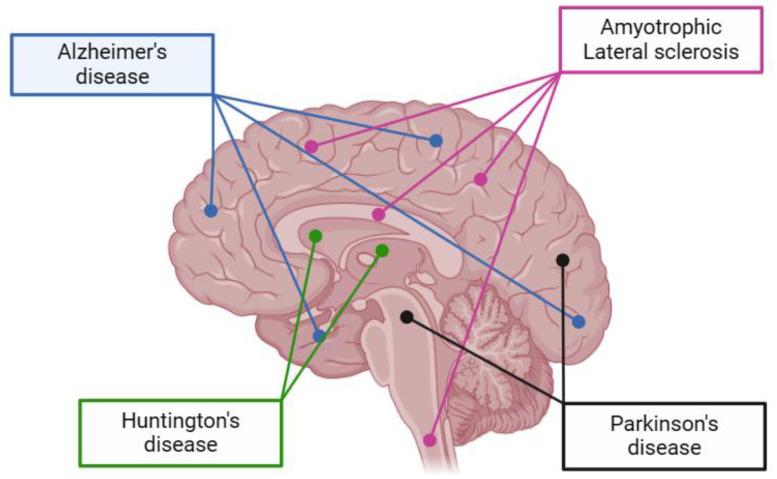
Neurodegenerative diseases associated with regions of the brain.

Figure 3 presents a visualization of the main neurodegenerative diseases and their affected regions in the brain.

AD primarily affects the hippocampus and its connected structures, making it harder for individuals to form new memories or learn new information. The hippocampus is responsible for retrieving short-term memories. As AD damage spreads throughout the brain, additional areas and lobes become affected, leading to problems with semantic memory and language as well as difficulties with recognizing familiar faces and objects [74,75]. In AD, the main pathological proteins are amyloid plaques and neurofibrillary tangles. Amyloid plaques are formed by the β-amyloid protein, especially the β-amyloid 42 form, which is toxic to brain cells. Neurofibrillary tangles consist of abnormal accumulations of the τ protein, which detach from normal microtubules and aggregate into structures that block the neuronal transport system. This results in cellular dysfunction and disruption of communication between neurons in AD [76,77].

PD is associated with damage to the basal ganglia, a group of small structures located deep in the subcortex that are primarily involved in motor control [78]. Lesions in this brain region are common in conditions of dementia that also present motor disturbances, such as PD dementia, Lewy body diseases, and Huntington’s disease dementia [79,80]. In PD and Lewy body dementia, Lewy bodies are the main pathological proteins involved. Lewy bodies are seen in the cerebral cortex, limbic system, and brainstem [77]. In dementia with Lewy bodies (DLB), early damage is seen in visual pathways and, in some cases, in the frontal lobes [81].

HD is a genetic disorder that affects the basal ganglia and leads to progressive degeneration of nerve cells in the brain, characterized by psychiatric disturbances, declining intelligence quotient, and uncontrollable motor dysfunction, resulting in progressive dementia and eventual death [82]. In HD, the main pathological protein involved is huntingtin, which is a protein encoded by the HTT gene. In people with HD, the huntingtin protein contains an abnormal repetition of the amino acid glutamine, which leads to progressive degeneration of nerve cells in the brain [83].

ALS is a severe neurodegenerative disease that affects the motor system of the human body, typically beginning in advanced stages of life. The deterioration of motor neurons occurs both in the cerebral motor cortex and in the brainstem motor nuclei, as well as in the anterior horns of the spinal cord [82]. In ALS, the main pathological proteins involved are superoxide dismutase 1 (SOD1), TAR DNA-binding protein 43 (TDP-43), and fused in sarcoma (FUS). These proteins are implicated in the degeneration of motor neurons, leading to muscle weakness, paralysis, and eventually respiratory failure [77].

In this context, efforts to develop therapeutic alternatives are on the rise, meeting consumer expectations for safety, effectiveness, and sustainability in the treatment of symptoms associated with these conditions.

The use of medicinal plant extracts such as *Harpagophytum procumbens* and *Turnera subulata* for the treatment of neurodegenerative diseases is one of these alternatives. However, it faces various challenges in ensuring the stability of active compounds and their delivery to the central nervous system. To optimize the use of these bioactive compounds, some challenges have been reported by Gorantla [84], and Wang et al. [72], including

1. Reduction in systemic inflammation: This involves neutralizing free radicals and suppressing proinflammatory cytokines in the body’s periphery, which, in turn, alleviates brain inflammation by acting on the BBB.
2. Enhancement in BBB integrity: This aims to prevent the brain inflammation associated with increased BBB permeability.
3. Ensuring penetration into the brain parenchyma: Enabling the targeting of glial cells to reduce brain inflammation.
4. Gastrointestinal protection and regulation of the gut–brain axis: This includes minimizing limitations such as solubility, chemical instability, bitter taste, and unpleasant odor, as well as overcoming gastrointestinal membrane barriers, the influence of other chemical components, and gastric residence time.

These challenges need to be addressed to fully explore the potential of medicinal plant extracts in the treatment of neurodegenerative diseases. Therefore, the study of specific delivery systems for plant extracts in the treatment of neurodegenerative diseases is a necessity due to the complexity of active compounds and the importance of optimizing bioavailability. Such systems aim to improve absorption, ensure compound stability, and minimize side effects, resulting in more precise and effective treatments for these debilitating conditions.

Nanotechnology has been applied to enhance the permeability, solubility, and stability of bioactive compounds. This enables a higher efficiency in delivering bioactive compounds to various target sites, including the brain, which requires bioactive molecules to overcome the BBB, which has a diameter of less than approximately 20 nm, restricting the passage of most bioactive molecules [85].

A wide variety of nanomaterials have been extensively explored to assist with the transport of small pharmacological molecules or therapeutic biomolecules to the central nervous system using polymeric nanomaterials, lipid-based carriers, polyesters, inorganic materials, and those coupled with cell membranes. The size of these nanomaterials is adapted according to the particles to be transported, having a diameter of approximately less than 200 nm. Additionally, other aspects are related to these systems, such as surface charge, topology, and shape [86,87].

Recent research has focused on innovative noninvasive lipid-based nanomaterial studies, such as solid lipid nanoparticles (SLNs) and nanostructured lipid carriers (NLCs), as they offer a range of advantages in terms of toxicity. These lipid-based carriers are less toxic when compared to polymeric ones, biodegradable, biocompatible, and capable of crossing brain barriers through diffusion. These advantages easily enable these encapsulated substances to reach their sites of action [86].

In nutrition and medicine, various nanorelease systems, such as nanoemulsions, nanoliposomes, and lipid or polymeric nanoparticles, are being investigated as functional carriers [88]. Furthermore, the surface modification of nanocarriers to include ligands targeted to receptors and transporters in the BBB enables overcoming receptor-mediated endocytosis, resulting in improved selectivity and permeability to the central nervous system [89].

Regarding the nanotechnologies used to date for the delivery of plant extracts and nutraceuticals, nanoemulsions are nanoscale emulsified systems designed to enhance drug delivery to the target site while minimizing adverse effects and toxic reactions. They consist of two immiscible liquid phases: a dispersed phase and a continuous phase. The stability of this system is achieved using emulsifying agents, including surfactants and co-surfactants. Particles in the submicron range, ranging from 10 nm to 1000 nm, act as vehicles to transport drug molecules to the desired site of action [90].

Gabal et al. [91] investigated the effect of the surface charge of the nanocarrier on the in vivo brain delivery of a hydrophilic drug via the nasal route and obtained ideal anionic and cationic nanostructured lipid carriers with a small particle size (<200 nm) that can be efficiently absorbed by brain tissue. Furthermore, the in situ gel of the anionic nanostructured lipid carrier provided the highest drug-targeting efficiency in the brain.

The feasibility of co-encapsulating two polyphenols, resveratrol and curcumin, in a mucoadhesive lipid nanoemulsion as a carrier system to improve their brain delivery for the transnasal treatment of neurodegenerative diseases was investigated by Nasr [92]. In this study, Nasr showed that the nanoemulsion was able to preserve the antioxidant capacity of the polyphenols and protect them from degradation. Furthermore, the mucoadhesive nanoemulsion was safe for the nasal mucosa and managed to increase the amounts of both polyphenols in the brain.

Another promising strategy for delivering bioactive compounds to the brain involves the use of lipid carriers known as liposomes. This lipid-based approach is notable due to its ability to replicate the natural lipid environment found in biomembranes, including the BBB. Moreover, liposomes play a vital role in protecting bioactive molecules from degradation during transport to their sites of action, reducing toxicity, and increasing biocompatibility compared to direct administration [92].

Saffari et al. [93] studied a preparation of phosphatidylserine nanoliposome formulations containing metformin and evaluated the therapeutic effectiveness of the new formulation in recovering memory impairment similar to that of AD in a rat model. The results of the study indicated significant reductions in inflammatory and necrotic neural cells, along with a significant increase in neurogenesis, in rats treated with the nanoliposome formulation. It also showed that the nanoliposome formulation could potentially be more effective than the free form of metformin in the recovery process of rats with AD [93].

Recently, PEGylated nanoliposomes containing rosemary extract were studied for the potential treatment of AD [94]. The results of the study indicated that optimized rosemary-extract-loaded nanoliposomes have significant potential for successful transport through the BBB and, therefore, provide an efficient and safe treatment for AD.

On the other hand, polymeric nanoparticles consist of a reservoir system and a matrix system that can be synthesized from natural or synthetic polymers. This is achieved through techniques such as emulsification–diffusion, nanoprecipitation, and solvent evaporation. This technology allows polymeric materials to encapsulate photochemical compounds, offering protection against external degradation and resulting in increased long-term storage stability. Additionally, these nanoparticles provide greater bioavailability and enable sustained and controlled release of the encapsulated compounds [95,96].

In the study conducted by Alberti and Maraschin [97], a project was developed to create nanoparticles containing β-caryophyllene for the controlled release of CB2 cannabinoid agonists. The work was conceived as a strategy to combat neurodegeneration. The results revealed that the nanoparticle formulation was effective for intranasal administration, demonstrating stability for up to a month.

It is known that curcumin has potential use in AD due to its antioxidant, antiamyloid, and anti-inflammatory activity. Nanoparticles (PLGA-PEG) conjugated with peptide B6 and curcumin, targeting the brain, were studied in HT22 cells and APP/PS1 AI transgenic mice [98]. The results of the study suggested that these nanoparticles could reduce the diameter of curcumin, thus increasing its cellular uptake while showing good blood compatibility. Additionally, the study of APP/PS1 mice showed an improvement in memory and spatial learning ability, indicating the promising potential of PLGA-PEG nanoparticles conjugated with peptide B6 and curcumin [98].

Despite the growing interest in applying nanotechnology for effective delivery of phytochemicals to the brain in the treatment of neurodegenerative diseases, it is noteworthy that research involving nanoencapsulated extracts of *Harpagophytum procumbens* and *Turnera subulata* has been, thus far, scarce or nonexistent.

Although nanotechnology has shown potential for enhancing the bioavailability and targeted delivery of bioactive compounds to the central nervous system, the exploration of these two specific plants in a nanoencapsulated context lacks significant studies. This can be attributed to the wide range of phytochemical compounds present in these plants and the need to adapt encapsulation strategies to optimize efficacy. For example, the stability of nutraceutical release systems under physiological conditions is a critical factor in their effectiveness and safety.

Nanocarriers such as liposomes, niosomes, solid lipid nanoparticles (SLNs), and nanostructured lipid carriers (NLCs) are promising systems for the release of nutraceuticals due to their ability to enhance the stability, solubility, and bioavailability of these compounds [99]. However, the stability of these carriers under physiological conditions can be affected by various factors, including pH, temperature, enzymatic degradation, and interactions with biological components [99]. Additionally, when nanoparticles are exposed to complex biological matrices such as blood, cerebrospinal fluid, tears, aqueous humor, vitreous humor, and synovial fluid, they may undergo significant physicochemical changes. These changes include the formation of an “aqueous corona” around the nanoparticles, altering their surface properties and potentially eliciting immune responses, modifying their fate and toxicity [100]. However, research findings, in light of the present study, do not provide specific information regarding the stability of nutraceutical delivery systems based on *Harpagophytum procumbens* and *Turnera subulata* under physiological conditions. Therefore, our study revealed that further research and development in this area are crucial to optimize the delivery of bioactive compounds from *Harpagophytum procumbens* and *Turnera subulata*.

The potential for immune responses and nanoparticle carriers is another important consideration in the development of nutraceutical delivery systems. Nanoparticles can interact with the immune system in various ways. Nanoparticles can be specifically designed to target or evade interactions with the immune system, with such interactions considered beneficial when they can lead to various beneficial medical applications [101].

Nanoparticle-based delivery systems offer potential advantages for the site-specific delivery of drugs, peptides, bioactive compounds, and genes; improvement in in vitro and in vivo stability; and reduction in side effects. However, since nanoparticles are often taken up by the phagocytic cells of the immune system, there may be undesirable interactions between the nanoparticles and the immune system, leading to the production of pro-inflammatory cytokines and activation of immune cells [102]. These immune responses can affect the stability, efficacy, and safety of nutraceutical delivery systems. Therefore, it is important to carefully assess the potential for immune responses to nanoparticle carriers and design delivery systems that minimize these risks, especially in the development of nanostructured nutraceuticals for the treatment of neurodegenerative diseases based on plants such as *Harpagophytum procumbens* and *Turnera subulata*.

## 4. Impact of Nutraceuticals on Neurodegenerative Diseases

Neurodegenerative diseases are characterized by progressive neuronal dysfunction and the death of neurons in specific areas of the brain and spinal cord, with impacts in advanced stages extending to other organs. Numerous pieces of evidence suggest that oxidative stress plays a crucial role in the development of these disorders. This stress leads to damage of the brain tissue and triggers inflammation in affected areas through the activation of microglial cells and astrocytes, resulting in the production of inflammatory mediators such as cytokines, chemokines, and reactive oxygen and nitrogen species. These processes provoke mitochondrial dysfunction and an influx of intracellular calcium, caused by the increased expression of certain genes and the accumulation of abnormal proteins such as beta-amyloid peptides and neurofibrillary tangles in AD, and alpha-synuclein and Lewy bodies in Parkinson’s neurons [103].

Oxidative stress plays a significant role in the pathogenesis of AD and PD. It is characterized by an imbalance between the production of reactive oxygen species (ROS) and the biosystem’s capacity to detoxify, leading to damage to neural cells [104].

In AD, oxidative stress is associated with the accumulation of β-amyloid (Aβ) plaques and neurofibrillary tangles, which are the major pathological features of the disease. The oxidative stress triggered by reactive oxygen and nitrogen species in AD affects neuronal glucose and glutamate transport, impairs mitochondrial membrane potential, and reduces ATPase sodium-potassium activity, contributing to neuronal dysfunction and damage [105]. The accumulation of Aβ is associated with the induction of oxidative stress, especially through the formation of stable complexes with metals such as iron, zinc, and copper, which generate reactive oxygen species [106]. This resulting oxidative stress promotes excitotoxicity, mitochondrial dysfunction, and the activation of the signaling pathways important in AD pathology. Additionally, lipoproteins isolated from patients with AD may facilitate the production of nitric oxide and peroxynitrite in astrocytes, implicating other cellular components of the central nervous system in the interaction between oxidative stress and AD pathophysiology [105].

In PD, oxidative stress has been associated with the degeneration of dopaminergic neurons. Disruption of redox homeostasis can lead to cell death, with oxidative and nitrosative damage identified in the substantia nigra. Dopamine, upon auto-oxidation, generates reactive oxygen species and dopaminergic quinones, contributing to oxidative stress in this brain region [107]. Mitochondrial dysfunction and oxidative stress are central components in the neurodegeneration in PD, along with defects in the clearance of abnormal protein aggregates, such as α-synuclein aggregation, which forms Lewy bodies rich in α-synuclein [108]. Aging reduces antioxidation capacity, decreasing the activity and expression of antioxidant genes such as SOD2, catalase, and glutathione peroxidase [109].

Parkinson’s disease is situated within the spectrum of Lewy body diseases, which includes diffuse Lewy body disease (DLBD) and dementia with Lewy bodies (DLB). In DLB, PD changes are observed along with the spread of Lewy bodies and Lewy neurites in the cerebral cortex and diencephalic nuclei [110]. Oxidative stress plays a crucial role in the pathogenesis of DLB. The presence of Lewy bodies, primarily composed of α-synuclein, is linked to increased levels of this protein and associated genetic mutations. Oxidative stress can induce α-synuclein aggregation, exacerbating DLB neurodegeneration. In addition to protein oxidation, lipid peroxidation is also a prominent feature of DLB. Highly peroxidizable docosahexaenoic acid is increased in the amygdala and frontal cortex in DLB, leading to increased lipoxidative damage. This damage is associated with the increased expression of advanced glycation end products (AGEs) and their receptors (RAGE) in the frontal cortex, amygdala, and substantia nigra. These alterations are not associated with α-synuclein aggregation in the cortex, suggesting that other factors associated with aggregation may also contribute to the neurodegeneration observed in DLB [111].

Antioxidant and anti-inflammatory agents have been proposed as potential therapeutic targets for both AD and PD, with the ability to neutralize reactive oxygen species (ROS), reduce oxidative stress, and affect immunological and inflammatory responses, which may help to slow down disease progression [104]. This is because the currently available treatments for these conditions are largely palliative, unable to effectively halt neuronal degeneration. In this context, the bioactive compounds found in plants have sparked interest due to their significant potential in relieving cognitive symptoms and treating neurodegenerative diseases in humans.

**Figure 4:**
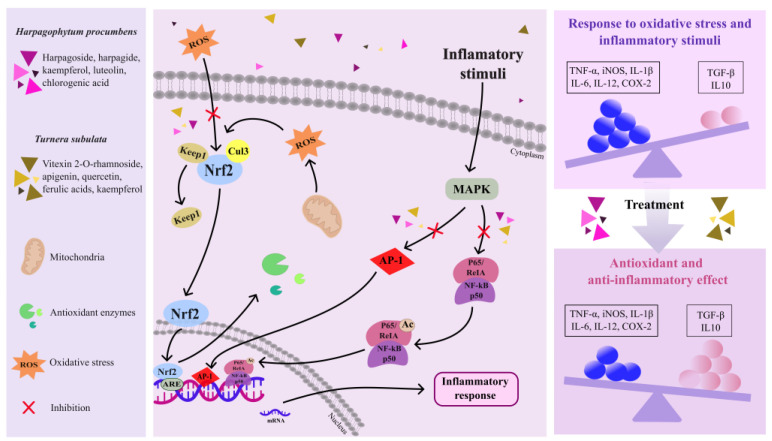
Potential mechanisms of action of anti-inflammatory and antioxidant molecules present in *Harpagophytum procumbens* and *Turnera subulata*. The specific pathways depicted represent only a subset of the numerous pathways involved in the inflammatory and oxidative processes occurring in neurodegenerative diseases.

Figure 4 illustrates the potential molecular mechanisms by which the bioactive compounds found in *Harpagophytum procumbens* and *Turnera subulata* may protect against neurodegenerative damage, specifically targeting the Nrf2 and NF-kB signaling pathways.

*Harpagophytum procumbens* and *Turnera subulata* are botanical species under investigation for the treatment of these diseases, with their mechanisms of action remaining incompletely elucidated. However, a study revealed that the ethanolic extract of *Harpagophytum procumbens* can inhibit inflammatory processes, reducing the release of cytokines and PGE2. Additionally, it also regulates the expression of genes related to inflammation, such as nRNA and COX-2, IL-6, and TNF-α. Furthermore, this extract demonstrated the ability to block the induction of proinflammatory genes, possibly by interfering with the AP-1 pathway [23]. Moreover, reports suggested that extracts of *Harpagophytum procumbens* can prevent lipid peroxidation caused by well-known pro-oxidants, as well as reduce the oxidative stress and loss of cell viability in brain homogenates induced by SNP or iron [112].

Harpagoside, one of the main compounds present in *Harpagophytum procumbens*, was associated with reduced lipid peroxidation and the increased activity of antioxidant enzymes such as superoxide dismutase and catalase [40]. Flavonoids like luteolin, which possess strong anti-inflammatory activity, function by inhibiting NF-kB activation, thereby reducing the expression of genes that promote inflammation and consequently the generation of inflammatory cytokines. Additionally, luteolin exhibits protective action on DNA against free radicals produced by H_2_O_2_ [113].

Regarding *Turnera subulata*, studies revealed that the aqueous and hydroalcoholic extracts of the flowers and leaves of this plant demonstrated the ability to modulate the inflammatory response in vitro. They influenced the secretion of pro-inflammatory cytokines (TNF-α, IL-1β, and IL-6) and anti-inflammatory cytokines (IL-10) and inhibited the production of PGE-2 and NO. These effects may be related to the presence of phytochemicals such as vitexin-2-O-rhamnoside and the synergy among various bioactive compounds in these extracts [62]. In a study conducted by Souza et al. [61], it was observed that an extract of *Turnera subulata* leaves demonstrated the ability to reduce oxidative stress in RAW-264.7 macrophage cells resulting from an inflammatory response. They highlighted that the extract could directly modulate the inflammatory response, affecting the activity of members of the mitogen-activated protein kinase pathways.

Both *Harpagophytum procumbens* and *Turnera subulata* contain antioxidant compounds, such as chlorogenic acid, which neutralize reactive oxygen species (ROS) to protect cells from oxidative stress [114]. Chlorogenic acid has demonstrated the ability to mitigate the generation of reactive oxygen species resulting from inflammatory agents such as tumor necrosis factor-alpha (TNF-α) and nitric oxide (NO). Furthermore, chlorogenic acid can regulate the expression of genes related to the antioxidant response, aiding cells in surviving oxidative stress [115]. Therefore, enzymes catalyzing the reduction of peroxides, such as hydrogen peroxide, using glutathione as a substrate, are encoded by genes like glutathione peroxidase (GPx), superoxide dismutase (SOD), and catalase (CAT) [116,117]. Thus, by increasing GPx expression, chlorogenic acid, along with the other flavonoids present in plants, may enhance the cell’s ability to remove peroxides and decrease oxidative stress [118].

Moreover, by binding to the antioxidant response element (ARE), genes regulating the Nrf2-ARE pathway govern the expression of additional genes involved in phase II xenobiotic metabolism and antioxidant defense mechanisms [114,119]. The activation of the Nrf2-ARE pathway can be initiated by flavonoids, which enhance the transcription of the genes implicated in oxidative stress mitigation and promote cellular survival [115,120].

## 5. Nutraceuticals from *Harpagophytum procumbens* and *Turnera subulata* in Neurodegenerative Diseases: Challenges and Future

The studies presented thus far regarding the use of nutraceuticals derived from *Harpagophytum procumbens* and *Turnera subulata* have demonstrated promising positive effects in the treatment of neurodegenerative diseases. However, it is important to note that the evidence is still limited, and there are gaps in the scientific research.

Further research is needed to fully understand the mechanisms of action and the effectiveness of these extracts in the treatment of neurodegenerative diseases. Many initial studies suggesting the benefits of these extracts have been conducted in animal models or in vitro, which may not necessarily reflect the complexity of neurodegenerative diseases in humans. Therefore, clinical studies in humans are essential to confirm the safety and efficacy of these therapeutic approaches. Additionally, understanding the mechanisms by which these extracts act in the treatment of neurodegenerative diseases is crucial for developing more targeted and effective treatments.

Both *Harpagophytum procumbens* and *Turnera subulata* contain a wide variety of bioactive compounds. Therefore, identifying which of these compounds are responsible for the beneficial effects and how they interact remains a challenge. Further studies in this field can aim to standardize and improve the quality of these extracts, which can vary significantly between different sources, making it difficult to compare results and verify consistency in treatments. Furthermore, standardizing and elucidating compound interactions is essential for assessing the long-term safety of using these nutraceuticals, as well as potential side effects.

The research on delivery systems for these extracts is scarce and can contribute significantly to advancing the research on these extracts. Given the therapeutic potential that these plants can offer in the context of neurodegenerative diseases, additional research must explore the application of nanotechnology for the delivery of their phytochemicals to the brain. These studies can provide valuable insights into the effectiveness of nanoencapsulation and the therapeutic impact of these approaches, thereby contributing to the advancement of the treatment of these debilitating diseases.

## 6. Conclusions

This literature review addressed the key therapeutic implications of using *Harpagophytum procumbens* and *Turnera subulata* in the context of neurodegenerative diseases, highlighting the promise of these nutraceuticals and the challenges ahead. The research has revealed that extracts from these plants have the potential to modulate inflammatory responses and reduce oxidative stress, two fundamental processes for the treatment of neurodegenerative diseases. However, the evidence is currently predominantly based on preclinical studies, making the transition to clinical trials crucial. Additionally, it is essential to consider nutraceutical delivery systems. Precise formulation and administration strategies play a critical role in the therapeutic efficacy of these extracts. The research and development of state-of-the-art delivery systems, such as nanoparticles and microparticles, can improve the bioavailability and controlled release of the bioactive compounds from these plants. Ultimately, the use of nutraceuticals derived from these extracts offers a promising therapeutic approach, but its full integration into clinical practice requires an ongoing commitment to research and interdisciplinary collaboration among scientists. The hope of alleviating the burden of neurodegenerative diseases is sustained by a future of innovative and dedicated research.

## Figures and Tables

**Table 1:** Some therapeutic properties associated with *Harpagophytum procumbens* in relation to neurodegenerative diseases.

Therapeutic Property	Description	References
Antioxidant potential	The compounds found in *Harpagophytum procumbens*, such as iridoid glycosides and phenolics, possess antioxidant properties that may help reduce oxidative stress, a factor associated with neurodegenerative diseases.	[40,41]
Possible inflammation reduction	The plant’s anti-inflammatory properties can aid in reducing chronic inflammation, which plays a role in the progression of neurodegenerative diseases such as Alzheimer’s and Parkinson’s.	[16]
Potential pain relief	*Harpagophytum procumbens’s* ability to alleviate pain can be beneficial for patients with neurodegenerative diseases who often suffer from chronic pain, improving their quality of life.	[30,42]
Complementary use	While not a cure for neurodegenerative diseases, the plant can be used as part of a complementary treatment regimen to alleviate symptoms like the muscle stiffness and discomfort commonly seen in these disorders.	[43]

**Table 2:** Therapeutic properties of *Turnera subulata* and its relationship with neurodegenerative diseases.

Therapeutic Property	Description	References
Antioxidant activity	Reduces oxidative stress, protecting nerve cells against damage	[8]
GABA modulation	Regulates the neurotransmitter GABA, providing antianxiety and relaxation effects	[59,60]
Free radical inhibition	Effectively eliminates free radicals, reducing cell damage related to neurodegenerative diseases	[61,62]
Anti-inflammatory action	Reduces inflammation in the nervous system, decreasing the progression of neurodegenerative diseases	[62]
Antimicrobial activity	Combats infections that can worsen neurodegenerative conditions	[55,66,67]
Improved Cerebral Blood Flow	Increases blood flow to the brain, enhancing the supply of nutrients and oxygen	[71]
Potential for Memory Enhancement	May have a positive effect on cognition, including memory	[60]
Anxiolytic Properties	Reduces anxiety, alleviating psychological symptoms associated with neurodegenerative diseases	[57,58]

## Data Availability

Data sharing is not applicable.
